# Prevalence and Risk Factors Associated With Anemia in Adolescent Females From Rural Maharashtra, India: Findings From the MAS 2 Project

**DOI:** 10.1155/anem/7015604

**Published:** 2025-06-05

**Authors:** Rohan Shah, Laila J. Tata, Andrew Fogarty, Agnieszka Lemanska, Pratyush Kabra, Anand Ahankari

**Affiliations:** ^1^Institute of Advanced Studies, University of Surrey, Guildford, UK; ^2^Department of Paediatrics, Dr. D. Y. Patil Medical College, Hospital and Research Center, Pune, India; ^3^Department of Social and Behavioral Sciences, Harvard T.H. Chan School of Public Health, Boston, Massachusetts, USA; ^4^Harvard T.H. Chan School of Public Health - India Research Center, Mumbai, India; ^5^Academic Unit of Lifespan and Population Health, School of Medicine, University of Nottingham, Nottingham, UK; ^6^Center for Perinatal Research, University of Nottingham, Nottingham, UK; ^7^Faculty of Health and Medical Sciences, University of Surrey, Guildford, UK; ^8^Department of Community Medicine, Dr Vaishampayan Memorial Government Medical College Solapur, Solapur, Maharashtra, India; ^9^School of Nursing and Public Health, Manchester Metropolitan University, Manchester, UK; ^10^Halo Medical Foundation, Andur, Maharashtra, India

## Abstract

**Background:** Anemia is a serious public health problem in India, affecting a large proportion of children, adolescent females, and women. The primary aim of the study was to investigate the prevalence and risk factors of anemia in adolescent females and to understand the feasibility of involving adolescent females from rural regions of Maharashtra through a combination of community-based recruitment and hospital-based investigation strategies.

**Methods:** A cross-sectional study was conducted involving unmarried adolescent females (10–19 years) from the Osmanabad district of Maharashtra (April–August 2018). Venous blood samples were taken, and anemia was defined using a hemoglobin cutoff of < 12.0 g/dL. Multiple logistic and linear regression models were used to explore associations of risk factors with anemia status and hemoglobin (Hb) levels, respectively.

**Results:** Out of 679 participants informed and invited to the study, data were available and analyzed for 401 (59.05%) participants. The prevalence of anemia was 29.42%. In the fully adjusted linear regression model, Hb levels reduced by 0.15 g/dL with each year increase in age (*β* = −0.15 [95% CI: −0.24 to −0.05], *p* = 0.002). Hb levels were lower in those engaged in paid work compared to those who were not (*β* = −1.19 [95% CI: −2.09 to −0.29], *p* = 0.010) and among those belonging to the Muslim religion (*β* = −0.75 [95% CI: −1.41 to −0.10], *p* = 0.024) compared to Hindus. In the fully adjusted logistic regression model, anemia likelihood increased significantly with age (OR: 1.24 [95% CI: 1.07–1.44], *p* = 0.004) and was higher in Muslims compared to Hindus (OR: 3.02 [95% CI: 1.14–7.99], *p* = 0.026). Pulses/lentils consumption (≥ 3 times a week) was associated with a decreased risk of anemia (OR: 0.51 [95% CI: 0.28–0.90], *p* = 0.022).

**Conclusion:** Using the World Health Organization criteria, the prevalence of anemia was moderately high among adolescent females in the study region. Comprehensive preventive measures for the adolescent female population are required, along with intervention programs that include education, nutrition, supplementation, and social support schemes.

## 1. Background

Anemia is a public health problem characterized by a reduction in the red blood cells or hemoglobin (Hb), which reduces the oxygen-carrying capacity of the blood. This condition can lead to fatigue, weakness, dizziness, and difficulty in breathing and may impact school performance and social–emotional development [1–5]. The recent Global Burden of Disease report stated that about 25% of the world's population is affected by anemia, which accounts for over 2 billion cases [6]. In 2019, anemia was responsible for over 50 million years of healthy life lost due to disability, where causes of anemia included dietary iron deficiency, thalassemia, sickle cell trait, and malaria [7]. A high prevalence is seen in children, adolescents, pregnant, and postpartum women. World Health Organization (WHO) reported that about 40% of children aged between 6 and 59 months and 30% of women of reproductive age (15–49 years) have anemia [8]. Adolescent females are at higher risk, especially in low- and middle-income countries (LMICs), with a higher burden in rural areas among poorer households [7]. In adolescents, anemia can negatively impact physical development during their critical development period, increase the risk of infection, and poor pubertal and neurocognitive growth [6]. Poor nutrition leads to micronutrient deficiency, particularly iron deficiency anemia, responsible for adolescent morbidity and mortality [7]. Adolescent health and well-being indicators from 195 countries and territories estimated that the prevalence of anemia among adolescent females was 40% in multiburden countries (188 million people out of 467 million), with India standing second (54.1%) after Bhutan (58.0%) [9].

Adolescents now account for 1.8 billion worldwide, of which, 90% live in LMICs [10]. The Government of India has been implementing several initiatives through the National Health Mission (NHM), which has a component to provide weekly iron and folic acid supplementation (WIFS) as an intervention to decrease the prevalence of adolescent anemia [11]. Despite continuous efforts by the Government, prevalence remains much higher in India. According to the recent National Family Health Survey (NFHS) of India, the prevalence of anemia in adolescent females 15–19 years of age increased from the NFHS-4 (2015–2016) to NFHS-5 (2019–2021) from 55.8% to 59.1%, and for adolescent males, it increased from 30.2% to 31.1% [12]. The Comprehensive National Nutrition Survey (CNNS) from India showed that 28.5% of adolescents (females: 39.6%, boys: 17.6%) were anemic. Hemoglobinopathies, iron deficiency, zinc, and vitamin A deficiencies were associated with an increased risk of anemia [13].

Maharashtra, India's second most populous state, has a high adolescent population, wherein every fifth person is between 10 and 19 years of age [14]. Anemia prevalence ranges from 28% to 68% among adolescents across regions of Maharashtra [15–18]. One of our previous community-based cross-sectional studies conducted in rural areas (of the Marathwada region of Maharashtra) found the prevalence to be as high as 87% among adolescent females (13–17 years) [19], demonstrating the need for further research in this region. Various studies in community settings in India including our outlined survey have mainly chosen the capillary Hb measurement method due to its feasibility in field areas [19, 20]. However, anemia assessment using venous blood samples conducted in hospital settings is preferred, considering they provide accurate measurements (gold standard) [21, 22]. There are no studies from the Marathwada region (∼19 million population), where the rural and difficult-to-reach adolescent population were involved in anemia research having venous blood investigations. Due to the limited infrastructure and lack of resources, field-based venous blood collection is challenging to conduct due to further required sample transportation and investigation facilities. Therefore, this study was designed to investigate the prevalence and risk factors of anemia in adolescent girls and to understand the feasibility of involving adolescent females from rural regions of Maharashtra to participate in research through a combination of community-based recruitment and hospital-based investigation strategies.

## 2. Methods

### 2.1. Study Context and Field Area

The Maharashtra Anemia Study Phase 2 (MAS 2) was initiated by Halo Medical Foundation (HMF), India, in collaboration with the University of Nottingham, United Kingdom. This was a cross-sectional study across 20 villages (population ∼40,000) located in the Osmanabad (Dharashiv) district of Marathwada region of the Maharashtra state of India. This region of India is considered as one of the country's underprivileged areas due to limited development, inadequate infrastructure, and frequent droughts. All study villages had similar challenges such as limited healthcare access, poor transport facilities, and lack of water purification infrastructure. Government nurses generally visit monthly, offering healthcare services focused primarily on pregnant women and vaccinations. Females qualifying for inclusion in the study were aged 10–19 years, unmarried, and residents of the study area and willing to come to the research site (located in the HMF Hospital) for participation.

### 2.2. Ethical Consideration

The ethical approval for the study was obtained from the Medical School Ethics Committee of the University of Nottingham, United Kingdom (Reference: 145–1707), and the Institutional Ethics Committee of the Ashwini Rural Medical College, Hospital, and Research Center, Maharashtra, India (Reference: ARMCH/IECHR/12/2017). The study was conducted using the Guidelines for Good Clinical Practice (GCP) set by the International Council for Harmonization [23] and the ethical guidelines for Biomedical Research on Human Subjects suggested by the Indian Council of Medical Research [24]. Participation in the study was only possible when an adolescent participant (< 18) was accompanied during their visit to the hospital by a family member such as one of the parents, a sibling above 18 years of age, or a guardian. This requirement was part of the ethical considerations, as the study population comprised of minors (< 18 years), who were required to travel to the research site. Those who were 18 or 19 years were allowed to provide consent for their own participation and were allowed to attend the hospital visit unaccompanied. In addition, in line with ethical considerations and the National Iron + Initiative guidelines for controlling iron deficiency anemia, participants who were found to be anemic were referred to a medical doctor at the HMF Hospital and provided with a complete blood count (CBC) report. After evaluation by a medical doctor, the physician prescribed 60 mg of elemental iron for 3 months, which was provided free of cost from the hospital. Participants were offered follow-up services at no cost at the hospital, ensuring their well-being and the integrity of the study.

### 2.3. Participant Recruitment and Data Collection

A health worker appointed by the HMF facilitated the recruitment of the project in each village. These health workers had worked with HMF on public health and development projects and were specially trained on procedures for this study. The recruitment and data collection period was from April 2018 to August 2018 (project duration: April 2017–March 2019). A full-time research coordinator was employed to plan and implement all project and research activities in collaboration with the network of village health workers. Between January and April 2018, community-level meetings across 20 villages were conducted to disseminate information about the project. These meetings were held mainly on Sundays, school holidays, and evenings to maximize attendance. Eligible participants willing to participate in the study were enlisted after meetings (unmarried, adolescent females aged 10–19 years). Health workers prepared a list of eligible participants from their allocated village and initiated contact with all eligible residents, assisted by the research coordinator during field visits. Those eligible were invited to the research site (located in the HMF Hospital) to participate in the study. All eligible participants were approached only once, and those who declined/did not inform their decision were not approached a second time. Participants were asked to register with their village-based health worker. The health worker planned a research visit through discussions with the research coordinator. The research coordinator and health workers did not select participants; instead, participation was by self-selection through registration. Participants were verbally informed about the project during village meetings and in written format.

During the visit to the research site, all information regarding the study was presented to each participant and accompanying adult, both verbally and in written format in the local language (Marathi). Written informed consent was obtained from each participant, and their accompanying adult and the research coordinator countersigned it. Due to logistic constraints, the sample size in the study was capped at ∼400 adolescent females. This limit was decided considering available funding ($12,000) and timeframe due to the nature of the MAS 2 project having both research and health service (charity) components. Participation in the study was entirely voluntary. All services at the HMF Hospital were provided at no cost to participants across the project duration. Participants and their guardians had to travel to the research site independently, and no travel compensation was provided for the visit due to limited financial resources.

Along with CBC, two additional investigations were conducted during the initial visit, namely, C-reactive protein (CRP) and ABO blood group typing. During follow-up visits (only in participants who were anemic and prescribed iron-folic acid [IFA]), they were checked for CBC. Research findings on systemic inflammation and association with Hb levels from the MAS 2 project are published elsewhere [25].

### 2.4. Sample Collection and Analysis

Following consent, all participants were interviewed using a validated questionnaire tool to collect information on sociodemographics, diet, anemia history, and anemia treatment. Anthropometric measurements were taken, including height, weight, and mid-upper arm circumference. This was followed by a venous blood withdrawal performed by a phlebotomist in a supine position. All laboratory investigations were conducted at the HMF Hospital using routinely standardized equipment.

The CBC was performed using a cell counter, Sysmex XP100 (Sysmex Corporation, Japan). Weight was taken using an OMRON digital scale. Height and mid-upper arm circumference were taken using standardized measuring tapes. The study coordinator checked and validated all study tools and equipment on the first working day of each month throughout the data collection period. Monthly reports for study equipment, data collection progress, and overall project monitoring were prepared by the study lead (AA) in collaboration with the project coordinator and the local co-investigator (PK). The local co-investigator (PK) regularly visited the study sites to ensure adherence to good research practices and compliance with the study protocol and ethical guidelines. This paper is reported in line with the STROBE guidelines for cross-sectional studies (Supporting Information 1).

### 2.5. Statistical Analysis

Data analyses were performed using the Statistical Package for Social Sciences (SPSS) V.26.0 (IBM, 2020). Descriptive statistics were presented for all participants, those with anemia, and those without anemia within the study population, expressed as numbers (N) and percentages or median (25^th^ and 75^th^ centile). Variables were characterized into three groups, namely, individual, dietary, and sociodemographic. The chi-square test and Mann–Whitney test were applied to check whether the difference between anemia and nonanemia is statistically significant at a 5% level of significance.

**Figure 1:**
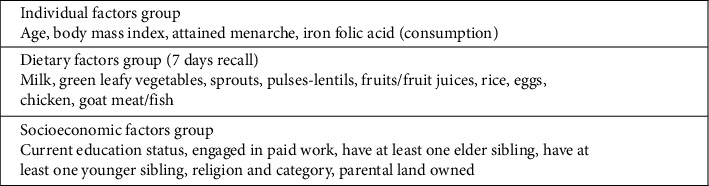
Risk factor groupings for multivariable modeling.

BMI was computed using the WHO's AnthroPlus software [26]. Based on Hb levels, anemia was categorized into four groups: no anemia (≥ 11.5 g/dL for ages 10–11, and ≥ 12.0 g/dL for ages 12–19), mild (11.0–11.4 g/dL for ages 10–11, and 11.0–11.9 g/dL for ages 12–19), moderate (8.0–10.9 g/dL for ages 10–19), and severe (< 8.0 g/dL for ages 10–19) [27]. Linear and logistic regression techniques were used to identify risk factors associated with adolescent anemia. Linear regression was used to assess the association between each risk factor and Hb level, and results were presented using regression coefficients (unstandardized β) with 95% confidence intervals (CIs). Logistic regression was used to assess the association of each risk factor with anemia status (severe/moderate/mild combined and compared with no anemia), and results were presented using odds ratios (ORs) and 95% CI. Initially, an unadjusted regression analysis was performed with all variables during both regression techniques. Other than age, all variables had subcategories. The 7-day dietary recall categories were simplified from four (never, 1–2 times a week, 3–6 times a week, everyday) to two (≤ 2 times a week, ≥ 3 times a week) to ensure an adequate number of participants in each category for regression analyses. After the unadjusted analyses, a multivariable analysis approach was used. In the adjusted analysis, risk factors were categorized into three groups based on their theoretical relationship with anemia and also based on the understanding of our field area: individual factors, dietary factors, and socioeconomic factors (Figure 1). Three multivariate models with mutual adjustment within groups were constructed, followed by a final model with full adjustment for all risk factors. Multicollinearity was examined using a covariate correlation matrix and variance inflation factor (VIF). Statistical significance was determined with a *p* value < 0.05. The above regression analyses' strategy was informed by our previous work through the MAS 1 project, which involved adolescent girls to study prevalence and risk factors associated with anemia [19].

## 3. Results

**Figure 2:**
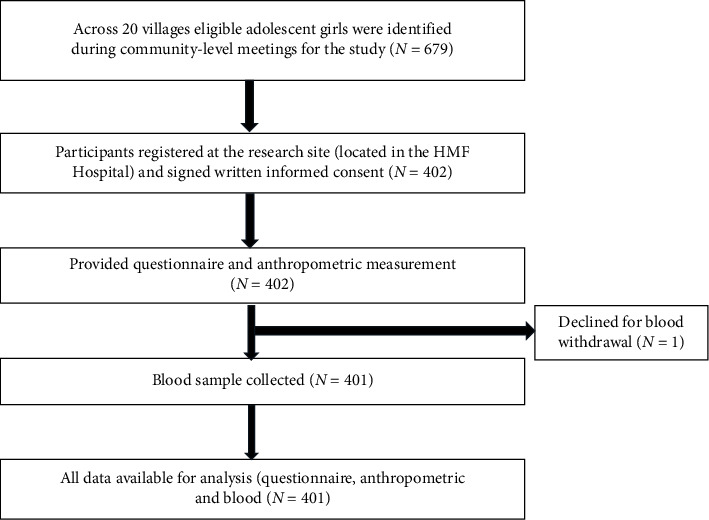
Flowchart of the recruitment process and final study population.

Of 679 eligible participants across 20 villages, 402 adolescent females accepted the study invitation and visited the research site based in the HMF Hospital, giving a response rate of 59.2%. One participant withdrew consent for a blood sample, which led to their exclusion from the study. Complete data, including blood investigations, were available from 401 participants (Figure 2). The anemia prevalence with Hb < 12.0 g/dL was 29.42%, with 1.74% identified with severe anemia (Hb < 8.0 g/dL for ages 10–19), 11.97% had moderate anemia (Hb: 8.0–10.9 g/dL for ages 10–19), and 15.71% had mild anemia (Hb: 11.0–11.4 g/dL for ages 10–11, and Hb: 11.0–11.9 g/dL for ages 12–19). None reported systemic disorders or conditions which could affect Hb levels. None reported blood transfusion within 12 months prior to enrollment in the study.

The participants were divided into two groups: those without anemia and those with anemia (Table 1). The median age of participants with anemia (15 years) was significantly higher than that of the nonanemic group (13 years), with an interquartile range of 13.00–17.00 and 12.00–15.00, respectively (*p* < 0.001).

Age, paid work, and Muslim religion (open and reserved category) were statistically significant in the fully adjusted linear regression model (Table 2). Hb levels reduced by 0.15 g/dL with a per year increase in age (*β*: −0.15 [95% CI: −0.24 to −0.05], *p* = 0.002). Similarly, Hb levels were lower in those engaged in paid work (some form of employment) (*β*: −1.19 [95% CI: −2.09 to −0.29], *p* = 0.01) and among those belonging to the Muslim religion (open and reserve category) (*β*: −0.75 [95% CI: −1.41 to −0.10], *p* = 0.024).

In the adjusted logistic regression model, the risk of anemia increased by 24% with a per year increase in age (OR: 1.24 [95% CI: 1.07–1.44], *p* = 0.003) (Table 3). Similarly, participants belonging to the Muslim religion (open or reserve category) were associated with a three times higher risk of having anemia compared to the Hindu religion (open category) (OR: 3.02 [95% CI: 1.14–7.99], *p* = 0.026). On the other hand, the risk of anemia was reduced by 49% if participants consumed pulses and lentils ≥ 3 times a week (OR: 0.51 [95% CI: 0.28 to 0.90], *p* = 0.022). The effect estimates of the studied risk factors showed consistency across all three models: unadjusted analysis, adjusted within the group, and fully adjusted model.

A total of 118 participants were found to be anemic (63 mild, 48 moderate, and 7 with severe anemia) and were referred for further medical advice. The participants were prescribed IFA supplements for 3 months, provided independently by the HMF Hospital, and availed from the hospital pharmacy without cost. Participants were advised by the doctor to return for a follow-up Hb estimation (CBC test) after 3 months. Of these 118, 67 (35 mild, 28 moderate, and 4 severe) returned for follow-up (follow-up rate 56.78%). The mean Hb was similar between the initial visit and follow-up visit post-IFA consumption (mean [SD]: 10.51 [1.70] versus 10.65 [1.76]; *p* = 0.171 [*N* = 67]). Of the 67 participants who attended follow-up visits, 41 had an increase in their Hb levels, 20 had a reduction, and 6 showed no difference in their Hb levels post-IFA consumption.

## 4. Discussion

### 4.1. Summary of Findings

The prevalence of anemia in our study was 29.42%, with most participants having mild (15.71%) and moderate anemia (11.97%) and a small proportion with severe anemia (1.74%). An increased risk of anemia was observed with increasing age, belonging to the Muslim religion, and engaged in paid work. In contrast, the risk was reduced with a higher intake of pulses and lentils (≥ 3 times a week).

### 4.2. Synthesis

Different parts of rural Maharashtra have reported varying prevalence ranging from 28% to 68% [15–18]. Similarly, other studies from rural India report high anemia prevalence rates between 46% and 70% [28–31]. Studies conducted in deprived areas have reported a prevalence of up to 90% [32] and a much higher prevalence of 96.5% among economically weaker groups compared to 65.18% in middle or higher economic groups [33]. The recent NFHS-5 project shows that more than 15 states have a high prevalence (> 55%) among socially backward groups [34]. The rural prevalence in other countries such as Turkey, Saudi Arabia, Iran, Palestine, and Egypt ranges from as low as 8.3%–46.6% [35–40].

The risk of anemia increased with age, consistent with previous studies on anemia from India [19, 41, 42]. Some of this elevated risk may be attributable to the age of menarche [43]. Research suggests that a shorter duration of menstrual bleeding is associated with high Hb levels, whereas heavy menstrual flow directly contributes to decreased Hb levels [43]. Similarly, the higher number of daily pad changes (a proxy for heavy menstruation) was found to be significantly associated with a higher risk of anemia (OR: 3.06 [95% CI: 1.60–5.84], *p* < 0.001) [44]. A study in rural western China involving young adolescent boys and girls (aged 10–14 years) reported an association between age and anemia even after adjusting for stages of puberty (*p* trend = 0.040) [45]. A study in Malaysian adolescent females showed an increasing trend of anemia across age groups (11.1% [95% CI: 6.7–17.8], 15.7% [95% CI: 11.4–21.3], and 23.1% [95% CI: 16.8–31.0]) at age 13, 15 and 17 years, respectively [46]. Similar findings were observed in young adolescents of Ethiopia, Sudan, and Tanzania, where higher prevalence was seen with an increase in age [47]. Contrary to our study, research conducted in Ethiopia reported females in early adolescence years (10–14) having a greater risk of anemia than those aged 15–17 and late adolescents 18–19 [48]. This may be due to differences in the onset of menarche across study populations or having participants, mainly from age 13–14, in the early adolescence group who may develop anemia after attaining menarche, as explained earlier.

Adolescents who engaged in paid work were associated with a higher risk of anemia. This could be attributed to various social and health causes outlined as follows. During crucial development stages across adolescence, nutritional demands are generally high. In our study area, adolescents from poorer families engage in work to support their family income. This mainly involves agricultural work, the primary income source in our study area. Adolescents engaged in paid work are likely to have higher nutrition demands due to physical activities; however, they are often not addressed due to limited food resources. Agricultural work and a poor diet have been described as a risk factor for chronic energy deficiency (CED) and anemia. Women performing agricultural work are 50% more likely to develop CED and anemia [49]. We understand that physical activity and poor diet may strain adolescent females during a period already requiring higher energy needs, leading to further deficiencies and anemia.

There was a higher risk of anemia in participants from the Muslim religion compared to Hindu religion participants. The findings are in agreement with previous studies from India and other LMICs. A study conducted by Agrawal and colleagues on young adolescents (10–19 years, both genders) in a coastal district of India reported higher odds of anemia in Muslims compared to those from the Hindu religion; however, it was not statistically significant (OR: 1.40 [95% CI: 0.82–2.43], *p* = 0.20) [50]. In another study performed on pregnant women in western Rajasthan (India), the proportion of anemia was higher in women belonging to the Muslim community (92.3%) compared to non-Muslim women (81.1%), respectively [51]. Findings from the Ethiopian Demographic and Health Survey on young women aged 15–24 years mentioned that anemia was more likely among Muslims compared to Orthodox Christians (aOR: 1.31 [95% CI: 1.07–1.70], *p* = 0.01] [52].

The participants in our study consumed less milk (≤ 2 times a week), green leafy vegetables, sprouts, fresh fruits or fruit juices, eggs, chicken, and goat meat/fish. Intake of pulses and lentils was associated with decreased odds of anemia (OR: 0.51 [95% CI: 0.28–0.90], *p* = 0.022]. Lentil is a good source of protein and is predominantly rich in albumin and globulin. DellaValle and team have reported that iron bioavailability in green lentils is far greater than in red lentils, even though the phytic acid values in green and red lentils are similar [53]. Lentils are the primary source of minerals for people on a vegetarian diet and contain some iron; however, their bioavailability is low. In a study, iron absorption from lentils was significantly lower than iron absorption from ferrous sulfate in iron-deficient women [54]. Pulses and lentil consumption was relatively higher in our population, where 77.81% of all participants consumed them ≥ 3 times a week. Nonetheless, there is limited evidence to investigate the association further observed.

### 4.3. Combination of Community-Based Recruitment and Hospital-Based Investigation Strategies

Our previous study (known as MAS 1) in a similar geographic area reported a very high prevalence of 87% in girls aged 13–17 [19]. One potential reason for such a high prevalence could be a difference in recruitment strategy and different methods of Hb estimation. The study also reported a similar association with age (identified as a risk factor), mid-upper arm circumference, and consumption of fruit (≥ 3 times a week), which were associated with a lower risk of anemia in this population [19]. We observed over 95% of the response rate in the MAS 1 project in the same region. MAS 1 involved 1010 adolescent females aged 13–17 years from 34 villages. Participants were approached within the community, and questionnaires and blood sample collection were made at the village level. Out of the 1035 individuals approached, 1010 participated in the study, resulting in a response rate of 98.00% [19]. Similarly, another study of the MAS 1 project targeted pregnant women between the third and fifth months of pregnancy from the same region. A total of 303 pregnant women were approached, and data collection was completed for 287 participants, yielding a response rate of 95% [55]. For our current study (MAS 2), based on consultation with key stakeholders, we incorporated community-based meetings to inform potential participants about the study, followed by hospital-based data collection and investigation strategies. This approach was specifically selected for us to understand the future scope of conducting hospital-based research where advanced blood investigations and long-term sample storage could be performed using venous blood samples. However, the response rate was 59.20%. Travel expenses for participants and their parent/guardian and remuneration to cover the loss of income for an accompanying family member could have improved our response rate. Though the response rate of our current hospital-based research work is much lower compared to our previous work in a village/community, it is moderate considering the size of the field area; the nearest village was 4 km, and the farthest was 50 km away from our research site (HMF Hospital). Our rural study population is often inaccessible and neglected in public health research. Thus, our study provides a valuable opportunity to inform future research and public health programs in anemia prevention and control, which may differ in risk factors from other areas. Nonetheless, it is plausible that those who were willing but could not afford to pay for their travel may have impacted our prevalence and associations observed.

### 4.4. Strengths and Limitations

A gold standard analytical method was used for hemogram (CBC) measurement using venous blood samples involving rural and difficult-to-reach adolescent populations. Laboratory equipment and tools had regular quality control checks across the study duration. Though the study setting is remote, an experienced research team led the data collection, ensuring strict adherence to research protocol and ethics procedures. To our knowledge, this is the first study from a rural Indian setting, where we have presented our learnings from a combination of community-based recruitment and hospital-based investigation strategies involving the adolescent girls' population. Our statistical analysis used two different but complementary approaches; the logistic regression analysis of anemia is clinically pertinent from a public health perspective; the linear regression analysis of Hb levels is more sensitive statistically to linear associations and hence helps maximize the value of this analysis using a clinical epidemiological approach. Due to limited funding resources, the sample size for this study was restricted as mentioned earlier. Our study area is much smaller, and findings may not be generalizable due to sociodemographic variations across Marathwada or Maharashtra state. Our study did not have access to resources such as modern laboratory equipment to measure iron, folate, vitamin B12, vitamin A, zinc, and helminth infestation, which are essential to investigate micronutrient deficiencies and causes of anemia in our population. The HMF Hospital is a secondary referral rural center with limited infrastructure, and due to budgetary constraints, it was not feasible to procure costly equipment or transport blood samples for 300 km to the nearest research center or a laboratory based in Pune city for such advanced blood investigations. A cross-sectional study approach prevents causal inference investigation considering the design limitations, and multiple regression can lead to Type 1 error(s). Dietary habits, anemia history, and menstrual history are prone to nondifferential and reporting biases. Some residual confounding is possible, although adjustments for several covariates were made. Our dataset had limited information about dietary practices; as a result, further investigations into higher risks in certain groups were not feasible. Considering our experience and understanding of the field area, we anticipate that this is likely to be due to access to food resources and lack of awareness, which needs further research. Considering the above issues, our study cannot provide specific recommendations about required interventions to address anemia; however, it provides an evidence to plan further work on anemia in rural India.

## 5. Conclusion

The prevalence of anemia among adolescent females in rural Maharashtra is moderately high. The key risk factors identified include age, engagement in paid work, Muslim religion, and low consumption of pulses and lentils. To address such multifactorial issues, comprehensive preventive measures are essential, encompassing education, nutrition, supplementation, and social support schemes for the entire adolescent female population. In addition, future research should focus on identifying modifiable/preventable causes of anemia in adolescents to plan effective interventional studies. Further research into medical and nonmedical causes of anemia is necessary to develop sustainable and holistic interventions to address this chronic public health issue in India. We understand that future projects involving a combination of community-based recruitment and hospital-based investigation models in a rural Indian setting would need financial support to enable participants to undertake travel as well as some remuneration to cover the loss of income for an accompanying family member.

## Figures and Tables

**Table 1:** Characteristics of study participants (*N* = 401).

Characteristics	All participants (*n* = 401)	No anemia (*n* = 283)	Anemia (*n* = 118)	*p* value
	*n* (%), percentage provided column-wise	*n* (%), percentage provided row-wise	*n* (%), percentage provided row-wise	
Individual factors group				
Median age^$^	14.00 (12.00, 16.00)	13.00 (12.00, 15.00)	15.00 (13.00, 17.00)	< 0.001
Age (years)				
10	28 (6.98%)	25 (89.29%)	3 (10.71%)	< 0.001
11	30 (7.48%)	28 (93.33%)	2 (6.67%)	
12	55 (13.72%)	40 (72.73%)	15 (27.27%)	
13	60 (14.96%)	49 (81.67%)	11 (18.33%)	
14	59 (14.71%)	40 (67.80%)	19 (32.20%)	
15	55 (13.72%)	36 (65.45%)	19 (34.55%)	
16	45 (11.22%)	26 (57.78%)	19 (42.22%)	
17	38 (9.48%)	20 (52.63%)	18 (47.37%)	
18	31 (7.73%)	19 (61.29%)	12 (38.71%)	
Body mass index (BMI)				
Underweight	73 (18.20%)	54 (73.97%)	19 (26.03%)	0.660
Healthy weight	304 (75.81%)	211 (69.41%)	93 (30.59%)	
Overweight + obese	24 (5.99%)	18 (75.00%)	6 (25.00%)	
Attained menarche				
Yes	276 (68.83%)	178 (64.49%)	98 (35.51%)	< 0.001
No	125 (31.17%)	105 (84.00%)	20 (16.00%)	
Age at the onset of menstrual cycle				
≤ 13	177 (64.13%)	118 (66.67%)	59 (33.33%)	0.313
≥ 14	99 (35.87%)	60 (60.61%)	39 (39.39%)	
School missed due to menstrual cycle				
Yes	41 (10.22%)	33 (80.49%)	8 (19.51%)	0.026
No	235 (58.60%)	147 (62.55%)	88 (37.45%)	
Not aware	125 (31.17%)	103 (82.40%)	22 (17.60%)	
Iron-folic acid supplementation (ever used)				
Yes	132 (32.92%)	87 (65.91%)	45 (34.09%)	0.151
No	269 (67.08%)	196 (72.86%)	73 (27.14%)	
Ever heard about anemia and hemoglobin tests				
Yes	194 (48.38%)	123 (63.40%)	71 (36.60%)	0.003
No	207 (51.62%)	160 (77.29%)	47 (22.71%)	
Checked hemoglobin in the recent 12 months				
Yes	70 (17.46%)	41 (58.57%)	29 (41.43%)	0.020
No	331 (82.54%)	242 (73.11%)	89 (26.89%)	
Told before that you are anemic				
Yes	26 (6.48%)	15 (57.69%)	11 (42.31%)	0.180
No	375 (93.52%)	268 (71.47%)	107 (28.53%)	
Dietary factors group				
Vegetarian (no egg)	137 (34.16%)	103 (75.18%)	34 (24.82%)	0.223
Vegetarian (consume egg)	63 (15.71%)	40 (63.49%)	23 (36.51%)	
Nonvegetarian	201 (50.12%)	140 (69.65%)	61 (30.35%)	
Milk consumption (in any form)				
Never	232 (57.86%)	162 (69.83%)	70 (30.17%)	0.242
1–2 times a week	84 (20.95%)	65 (77.38%)	19 (22.62%)	
≥ 3 times a week	85 (21.20%)	56 (65.88%)	29 (34.12%)	
Green leafy vegetables				
Never	135 (33.67%)	91 (67.41%)	44 (32.59%)	0.588
1–2 times a week	198 (49.38%)	142 (71.72%)	56 (28.28%)	
≥ 3 times a week	68 (16.96%)	50 (73.53%)	18 (26.47%)	
Sprouts				
Never	231 (57.61%)	156 (67.53%)	75 (32.47%)	0.292
1–2 times a week	163 (40.65%)	122 (74.85%)	41 (25.15%)	
≥ 3 times a week	7 (1.75%)	5 (71.43%)	2 (28.57%)	
Pulses and lentils				
Never	2 (0.50%)	2 (100.00%)	0 (0.00%)	0.082
1–2 times a week	87 (21.70%)	54 (62.07%)	33 (37.93%)	
≥ 3 times a week	312 (77.81%)	227 (72.76%)	85 (27.24%)	
Fruits/fruit juices				
Never	231 (57.61%)	156 (67.53%)	75 (32.47%)	0.292
1–2 times a week	163 (40.65%)	122 (74.85%)	41 (25.15%)	
≥ 3 times a week	7 (1.75%)	5 (71.43%)	2 (28.57%)	
Rice				
Never	11 (2.74%)	8 (72.73%)	3 (27.27%)	0.657
1–2 times a week	35 (8.73%)	27 (77.14%)	8 (22.86%)	
≥ 3 times a week	355 (88.53%)	248 (69.86%)	107 (30.14%)	
Eggs				
Never	287 (71.57%)	205 (71.43%)	82 (28.57%)	0.832
1 to 2 times a week	104 (25.94%)	71 (68.27%)	33 (31.73%)	
≥ 3 times a week	10 (2.49%)	7 (70.00%)	3 (30.00%)	
Chicken				
Never	320 (79.80%)	225 (70.31%)	95 (29.69%)	0.520
1–2 times a week	72 (17.96%)	53 (73.61%)	19 (26.39%)	
≥ 3 times a week	9 (2.24%)	5 (55.56%)	4 (44.44%)	
Goat meat/fish (meat other than chicken)				
Never	367 (91.52%)	260 (70.84%)	107 (29.16%)	0.117
1–2 times a week	28 (6.98%)	21 (75.00%)	7 (25.00%)	
≥ 3 times a week	6 (1.50%)	2 (33.33%)	4 (66.67%)	
Socioeconomic factors group				
Current education status (attending school/college)				
Yes	367 (91.52%)	261 (71.12%)	106 (28.88%)	0.433
No	34 (8.48%)	22 (64.71%)	12 (35.29%)	
Engaged in paid work				
Yes	18 (4.49%)	10 (55.56%)	8 (44.44%)	0.153
No	383 (95.51%)	273 (71.28%)	110 (28.72%)	
At least one elder sibling				
Yes	253 (63.09%)	174 (68.77%)	79 (31.23%)	0.301
No	148 (36.91%)	109 (73.65%)	39 (26.35%)	
At least one younger sibling				
Yes	285 (71.07%)	198 (69.47%)	87 (30.53%)	0.449
No	116 (28.93%)	85 (73.28%)	31 (26.72%)	
Religion and category				
Hindu open category	181 (45.14%)	134 (74.03%)	47 (25.97%)	0.098
Hindu reserve category (SC/ST/OBC)	194 (48.38%)	135 (69.59%)	59 (30.41%)	
Muslim open and reserve category	26 (6.48%)	14 (53.85%)	12 (46.15%)	
Parental land owned				
None	86 (21.45%)	59 (68.60%)	27 (31.40%)	0.738
Less than 5 acres	175 (43.64%)	127 (72.57%)	48 (27.43%)	
More than 5 acres	140 (34.91%)	97 (69.29%)	43 (30.71%)	
Parental livestock owned				
Yes	285 (71.07%)	205 (71.93%)	80 (28.07%)	0.350
No	116 (28.93%)	78 (67.24%)	38 (32.76%)	
Below poverty line (BPL) registered				
Yes	147 (36.66%)	101 (68.71%)	46 (31.29%)	0.570
No	249 (62.09%)	178 (71.49%)	71 (28.51%)	
Do not know (DN)	5 (1.25%)	4 (80.00%)	1 (20.00%)	
Parental house structure				
Temporary (kaccha)	1 (0.25%)	1 (100.00%)	0 (0.00%)	0.459
Semipermanent (semipucca)	352 (87.78%)	245 (69.60%)	107 (30.40%)	
Permanent (pucca)	48 (11.97%)	37 (77.08%)	11 (22.92%)	
Parental ownership of television				
Yes	289 (72.07%)	208 (71.97%)	81 (28.03%)	0.323
No	112 (27.93%)	75 (66.96%)	37 (33.04%)	
Parental ownership of mobile phone				
Yes	393 (98.00%)	277 (70.48%)	116 (29.52%)	0.781
No	8 (2.00%)	6 (75.00%)	2 (25.00%)	

*Note:* Values were presented as *n* (%), and *p* values were calculated using the chi-square test.

^$^Values presented as median (25^th^ and 75^th^ percentiles) had *p* values calculated using the Mann–Whitney test.

**Table 2:** Linear regression: risk factors associated with hemoglobin levels in adolescent females (*N* = 401).

Characteristics	Unadjusted β (95% CI)	Models adjusted for within-group factors only^a^ β (95% CI)	Fully adjusted models^b^ β (95% CI)
*Individual factors group*			
Age	−0.15 (−0.21 to −0.09)^∗∗^	−0.12 (−0.21 to −0.03)^∗^	−0.15 (−0.24 to −0.05)^∗^
Body mass index (BMI) (normal weight taken as reference)			
Underweight	0.05 (−0.33–0.43)	−0.16 (−0.56 to 0.23)	−0.08 (−0.49 to 0.32)
Overweight	0.00 (−0.62–0.63)	0.07 (−0.54–0.69)	0.13 (−0.49–0.75)
Attained menarche^#^			
Yes	−0.61 (−0.93 to −0.30)^∗∗^	−0.20 (−0.65 to 0.24)	−0.10 (−0.56 to 0.35)
Iron-folic acid (ever used)^#^			
Yes	−0.32 (−0.64 to −0.01)^∗^	−0.24 (−0.55 to 0.07)	−0.24 (−0.56 to 0.08)

*Dietary factors group (≤* *2 times a week taken as reference)*			
Milk consumption			
≥ 3 times a week	−0.16 (−0.53 to 0.19)	−0.14 (−0.51 to 0.21)	−0.29 (−0.65 to 0.07)
Green leafy vegetables			
≥ 3 times a week	0.23 (−0.16–0.63)	0.25 (−0.14–0.65)	0.35 (−0.03–0.74)
Sprouts			
≥ 3 times a week	−0.50 (−1.64 to 0.62)	−0.45 (−1.60 to 0.70)	−0.11 (−1.24 to 1.01)
Pulses and lentils			
≥ 3 times a week	0.01 (−0.34–0.37)	0.05 (−0.30–0.42)	0.08 (−0.28–0.45)
Fruits/fruit juices			
≥ 3 times a week	−0.11 (−0.44 to 0.20)	−0.11 (−0.44 to 0.21)	−0.07 (−0.40 to 0.25)
Rice			
≥ 3 times a week	−0.32 (−0.79 to 0.13)	−0.306 (−0.78 to 0.17)	−0.258 (−0.73 to 0.21)
Eggs			
≥ 3 times a week	−0.08 (−1.03 to 0.86)	0.00 (−0.96–0.97)	0.04 (−0.90–0.99)
Chicken			
≥ 3 times a week	−0.24 (−1.25 to 0.75)	−0.06 (−1.17 to 1.04)	0.04 (−1.04–1.13)
Goat meat/fish			
≥ 3 times a week	−0.38 (−1.60 to 0.84)	−0.27 (−1.62 to 1.07)	0.27 (−1.06–1.62)

*Socioeconomic factors group*			
Current education status (attending school taken as reference)			
School dropout	−0.33 (−0.87 to 0.19)	0.35 (−0.32–1.03)	0.68 (−0.0–1.37)
Engaged in paid work ^#^			
Yes	−0.97 (−1.68 to −0.26)^∗^	−1.14 (−2.04 to −0.24)^∗^	−1.19 (−2.09 to −0.29)^∗^
Have at least one elder sibling^#^			
Yes	−0.18 (−0.49 to 0.11)	−0.18 (−0.52 to 0.15)	−0.11 (−0.45 to 0.22)
Have at least one younger sibling^#^			
Yes	0.02 (−0.30–0.34)	−0.06 (−0.43 to 0.30)	−0.09 (−0.46 to 0.26)
Religion and category (Hindu open category taken as reference)			
Hindu reserve category (SC/ST/OBC)	−0.10 (−0.40 to 0.20)	−0.02 (−0.35 to 0.31)	−0.13 (−0.46 to 0.20)
Muslim open and reserve category	−0.65 (−1.27 to −0.03)^∗^	−0.65 (−1.30 to −0.00)^∗^	−0.75 (−1.41 to −0.10)^∗^
Parental land owned (no land category taken as reference)			
Less than 5 acres	0.30 (−0.09–0.69)	0.19 (−0.22–0.61)	0.24 (−0.17–0.66)
More than 5 acres	0.13 (−0.27–0.54)	−0.01 (−0.46 to 0.43)	0.03 (−0.41–0.48)

*Note: β* = linear regression coefficient.

Abbreviation: CI = confidence interval.

^a^Models with within-group adjustments include only risk factors within that group, that is, there were three models: the first was adjusted only for individual factors, the second adjusted only for dietary factors, and the third was adjusted for socioeconomic factors (see Figure 1 for risk factor groupings for multivariable modeling).

^b^The fully adjusted model includes all risk factors mentioned in the table.

^∗^ *p* < 0.05.

^∗∗^ *p* < 0.001.

^#^Taken NO as the reference category.

**Table 3:** Logistic regression: risk factors associated with anemia in adolescent females (*N* = 401).

Characteristics act	Unadjusted OR (95% CI)	Models adjusted for within-group factors only^a^ OR (95% CI)	Fully adjusted models^b^ OR (95% CI)
*Individual factors group*			
Age	1.26 (1.14–1.39)^∗∗^	1.18 (1.04–1.35)^∗^	1.24 (1.07–1.44)^∗^
Body mass index (normal weight taken as reference)			
Underweight	0.79 (0.44–1.42)	1.12 (0.60–2.09)	1.00 (0.51–1.97)
Overweight	0.75 (0.29–1.96)	0.68 (0.25–1.82)	0.68 (0.24–1.90)
Attained menarche^#^			
Yes	2.89 (1.68–4.95)^∗∗^	1.65 (0.80–3.42)	1.51 (0.70–3.24)
Iron-folic acid (ever used)^#^			
Yes	1.38 (0.88–2.17)	1.23 (0.77–1.97)	1.26 (0.75–2.10)

*Dietary factors group (≤* *2 times a week taken as reference)*			
Milk consumption			
≥ 3 times a week	1.32 (0.79–2.20)	1.34 (0.79–2.27)	1.72 (0.97–3.06)
Green leafy vegetables			
≥ 3 times a week	0.83 (0.46–1.50)	0.79 (0.43–1.45)	0.64 (0.34–1.23)
Sprouts			
≥ 3 times a week	0.95 (0.18–5.01)	0.75 (0.13–4.13)	0.45 (0.07–2.77)
Pulses and lentils			
≥ 3 times a week	0.63 (0.38–1.04)	0.58 (0.35–0.98)^∗^	0.51 (0.28–0.90)^∗^
Fruits/fruit juices			
≥ 3 times a week	1.50 (0.95–2.38)	1.53 (0.95–2.46)	1.51 (0.90–2.52)
Rice			
≥ 3 times a week	1.37 (0.67–2.80)	1.45 (0.69–3.03)	1.45 (0.66–3.16)
Eggs			
≥ 3 times a week	1.02 (0.26–4.04)	0.88 (0.20–3.83)	0.83 (0.18–3.84)
Chicken			
≥ 3 times a week	1.95 (0.51–7.39)	0.93 (0.19–4.46)	0.76 (0.14–4.07)
Goat meat/fish			
≥ 3 times a week	4.93 (0.89–27.29)	4.27 (0.64–28.35)	2.53 (0.33–19.49)

*Socioeconomic factors group*			
Current education status (attending school taken as reference)			
School dropout	1.34 (0.64–2.81)	0.69 (0.25–1.89)	0.36 (0.12–1.06)
Engaged in paid work^#^			
Yes	1.98 (0.76–5.16)	2.25 (0.63–7.99)	2.48 (0.64–9.54)
At least one elder sibling^#^			
Yes	1.26 (0.80–1.99)	1.35 (0.82–2.23)	1.29 (0.75–2.22)
At least one younger sibling^#^			
Yes	0.83 (0.51–1.34)	1.31 (0.76–2.25)	1.52 (0.84–2.72)
Religion and category (“Hindu open category” taken as reference)			
Hindu reserve category (SC/ST/OBC)	1.24 (0.79–1.95)	1.21 (0.73–1.99)	1.49 (0.87–2.56)
Muslim open and reserve category	2.44 (1.05–5.65)^∗^	2.56 (1.04–6.28)^∗^	3.02 (1.14–7.99)^∗^
Parental land owned (“no land” taken as reference)			
< 5 acres	0.82 (0.47–1.45)	0.93 (0.50–1.73)	0.85 (0.43–1.66)
≥ 5 acres	0.96 (0.54–1.73)	1.17 (0.60–2.25)	1.05 (0.52–2.14)

Abbreviations: CI = confidence interval; OR = odds ratio.

^a^Models with within-group adjustments include only risk factors within that group, that is, there were three models: the first was adjusted only for individual factors, the second was adjusted only for dietary factors, and the third was adjusted for socioeconomic factors (see Figure 1 for risk factor groupings for multivariable modeling).

^b^The fully adjusted model includes all risk factors mentioned in the table.

^∗^ *p* < 0.05.

^∗∗^ *p* < 0.001.

^#^Taken NO as the reference category.

## Data Availability

The data that support the findings of this study are available on request from the corresponding author. The data are not publicly available due to privacy or ethical restrictions.

## Supporting Information

Supporting information includes the STROBE guidelines for cross-sectional studies. The study is reported in line with the guidelines.
